# Overexpression of TMPRSS4 in non-small cell lung cancer is associated with poor prognosis in patients with squamous histology

**DOI:** 10.1038/bjc.2011.432

**Published:** 2011-11-08

**Authors:** L Larzabal, P A Nguewa, R Pio, D Blanco, B Sanchez, M J Rodríguez, M J Pajares, R Catena, L M Montuenga, A Calvo

**Affiliations:** 1 Laboratory of Novel Therapeutic Targets, Division of Oncology, Center for Applied Medical Research, University of Navarra, Pamplona, Spain; 2 Laboratory of Biomarkers, Division of Oncology, Center for Applied Medical Research, University of Navarra, Pamplona, Spain; 3 Department of Biochemistry, University of Navarra, Pamplona, Spain; 4 Department of Histology and Pathology, University of Navarra, Pamplona, Spain; 5 Research Department, Ingenasa, Madrid, Spain

**Keywords:** lung cancer, TMPRSS4, squamous cell carcinoma, serine protease

## Abstract

**Background:**

Mortality rates in lung cancer patients have not decreased significantly in recent years, even with the implementation of new therapeutic regimens. One of the main problems is that a large proportion of patients present local or distant metastasis at the time of diagnosis. The need for identification of novel biomarkers and therapeutic targets for a more effective management of lung cancer led us to investigate TMPRSS4, a protease reported to promote tumour growth and metastasis.

**Material and methods:**

In all, 34 lung cancer cell lines were used to evaluate the TMPRSS4 expression. Cell migration and clonogenic assays, and an *in-vivo* lung metastasis model were used for functional analysis of the TMPRSS4 downregulation in H358, H441 and H2170 cell lines. The TMPRSS4 expression analysis in normal and malignant lung tissue samples was performed by qPCR. Five different microarray-based publicly available expression databases were used to validate our results and to study prognosis.

**Results:**

The TMPRSS4 knock down in H358, H441 and H2170 cells resulted in a significant reduction in proliferation, clonogenic capacity and invasion. A significant (*P*<0.05) decrease in the lung colonisation and growth was found when mice were injected with TMPRSS4-depleated H358-derived clones, as compared with controls. Expression of TMPRSS4 showed a >30-fold increase (*P*<0.001) in tumours in comparison with non-malignant samples. Levels in tumours with squamous cell carcinoma (SCC) histology were found to be significantly higher (*P*<0.001) than those with adenocarcinoma (AC) histology, which was confirmed in data retrieved from the microarrays. Kaplan–Meier curves demonstrated that high levels of TMPRSS4 were significantly associated (*P*=0.017) with reduced overall survival in the patients with SCC histology, whereas no correlation was found for the AC histology.

**Conclusion:**

Our results demonstrate that TMPRSS4 has a role in the lung cancer development. The potential use of TMPRSS4 as a biomarker for lung cancer detection or as a predictor of patient's outcome warrants further investigation.

Lung cancer is a critical problem in public health. It represents the most frequent tumour type in men and the second in women, and the 5-year survival rate remains inferior to 20% (Jemal *et al*, 2010). Most of the lung cancer cases belong to the non-small-cell lung cancer (NSCLC) type (85% of them). Histological subtypes include adenocarcinoma (AC) and squamous cell carcinoma (SCC) accounting for 30 and 50% of NSCLC, respectively (Youlden *et al*, 2008). The ACs are malignant epithelial tumours with glandular differentiation, positive for the TTF1 marker. The SCCs are derived from bronchial epithelial cells and express keratins (CK5 and CK6) but not TTF1 (Langer *et al*, 2010). More than 60% NSCLC patients present locally advanced, unresectable or metastatic (stage III/IV) tumours at the time of diagnosis, which fatally concludes in death within few months after the diagnosis. Therefore, there is a need for identifying new potential therapeutic targets against which more effective treatments may be developed.

There is a growing evidence demonstrating the critical implication of several genes in cancer development and metastasis, that have been classified as the tumour initiation genes (such as *EGFR*, *KRAS* and *MYC*), metastasis initiation (*TWIST*, *SNAIL* and *SLUG*), progression (metalloproteinases, *LOX* and *ANGPTL4*) and metastasis virulence genes (*IL6*, *IL11* and *TNFα*) (Nguyen *et al*, 2009). Deregulation of proteases is considered to be a hallmark of cancer development, because malignant cells require a range of proteolytic activities to enable growth, survival, motility, invasion and digestion of the extracellular matrix (Roy *et al*, 2009). Different studies have indicated that matrix metalloproteinases (MMPs) and serine proteases have an important role in cancer invasion and metastasis (Duffy, 1996). It is well established that proteases contribute to the degradation of the basement membrane and extracellular matrix (Roy *et al*, 2009). This property allows the tumour cells to invade the surrounding tissue and nearby blood vessels, an essential step in the metastasis development (Gupta and Massague, 2006). Furthermore, MMPs can modulate cell adhesion and proliferation by allowing the bioavailability of growth factors or cell-surface receptors (Fowlkes *et al*, 1994). In spite of these demonstrated roles in preclinical studies, agents targeting MMPs exhibited poor performances in clinical trials (Chu *et al*, 2007; Gialeli *et al*, 2011).

Most members of the serine protease family are either secreted or sequestered in the cytoplasmic organelles. Recently, type II transmembrane serine proteases (TTSPs) that are directly anchored to the plasma membranes have been described (Netzel-Arnett *et al*, 2003). These proteases participate in the regulation of cellular signalling events at the plasma membrane and in the extracellular matrix (Hooper *et al*, 2001). Many of the TTSPs show restricted tissue distribution in normal cells, but their expression is increased during the tumour growth and progression (Benaud *et al*, 2002).

TMPRSS4 is one of the TTSPs that is upregulated in pancreatic cancer and has been suggested as a diagnostic marker for the malignant thyroid neoplasms (Kebebew *et al*, 2005). It was demonstrated that TMPRSS4 is implicated in cell invasion, migration and adhesion of cancer cells. Moreover, the overexpression of TMPRSS4 in colon cancer cells was shown to promote epithelial–mesenchymal transition (EMT) through the upregulation of integrin α5, thereby enhancing motility and invasiveness (Jung *et al*, 2008; Kim *et al*, 2010). Although preliminary studies using reverse transcription–PCR (RT–PCR) have described the upregulation of this serine protease in few lung cancer specimens compared with the normal tissues (Jung *et al*, 2008), its expression levels in relation to clinical outcome and its function in lung cancer development is still unclear. In the present work, the expression and biological role of TMPRSS4 were studied in the two principal subtypes of NSCLC. We demonstrate that this protease is implicated in the NSCLC proliferation and migration. Furthermore, we show that TMPRSS4 is highly expressed in tumours compared with the normal lungs, with a higher expression in SCC than in AC. Importantly, we also demonstrate that high TMPRSS4 mRNA levels in SCC are associated with poor prognosis.

## Material and methods

### Patients

Data from 1022 NSCLC patients were analysed in this study, which belonged to six different cohorts: A (*n*=30; 11 AC+18 SCC+1 sarcomatoid epidermoid carcinoma histology), B (*n*=111; 58 AC+53 SCC), C (*n*=171; 125 AC+46 SCC), D (*n*=138; 62 AC+76 SCC), E (*n*=130; all SCC) and F (*n*=442; all AC). Cohorts other than A corresponded to series of patients analysed by microarray techniques, which were previously published: B (Bild *et al*, 2006), C (Potti *et al*, 2006), D (Lee *et al*, 2008), E (Raponi *et al*, 2006) and F (Shedden *et al*, 2008).

Tumour samples in the group A were obtained from the NSCLC patients who underwent tumour resectional surgery at the Clinica Universidad de Navarra (Pamplona, Spain) and at the Hospital Marqués de Valdecilla (Santander, Spain). In all, 15 tumour samples from this set were obtained with their corresponding matched non-malignant lung tissues; part of the tumour was fixed in 10% buffered formalin and processed for paraffin embedding and the other portion was frozen for molecular analysis. A 4-*μ*m thick section from the paraffin-embedded tissues was cut and analysed by histology after hematoxylin and eosin (H&E) staining. Sections of frozen samples were cut in a cryostat and analysed histopathologically to ensure that more than 70% of the tumour samples contained malignant tissue before being used for RNA extraction. Clinical and pathological characteristics of this cohort are shown in Table 1. The study protocol was approved by the Ethical Committee of our institutions.

### Cell culture

All human lung cancer cell lines used in this study were obtained from the the American Type Culture Collection (Manassas, VA, USA) and were maintained in RPMI 1640 with 10% foetal bovine serum at 37 °C and 5% CO_2_.

H358 cells were transduced with the pSFG_NES_TGL retroviral vector as previously described (Ponomarev *et al*, 2004). Vector containing GFP+ cells were selected by fluorescent-activated cell sorting. More than 90% of the cells were confirmed to express the reporter construct.

### Transfection with short-hairpin RNA

H358, H441 and H2170 cells were transfected with scrambled short-hairpin RNA (shCtrl) or shRNA against TMPRSS4 (shTMP4), kindly donated by Dr Anna Ruiz (Biomedical Research Unit, Vall d’Hebron, Barcelona, Spain). Lipofectamine 2000 (Invitrogen, Carlsbad, CA, USA) was used to perform transfections according to the manufacturer's instructions. After 24 h, cells were treated with 1 mg ml^−1^ hygromycin (Invitrogen) for 15 days to select the vector-containing cells. After analysis of TMPRSS4 mRNA levels by RT–PCR, clones of H358 (shTMP4-2 and shTMP4-3) and H2170 cell line (shTMP4-8) and H441 cell pools (shTMP4) were isolated, expanded and subjected to proliferation, clonogenic and migration assays.

### RT–PCR

Total RNA was isolated using the RNEasy Minikit (Qiagen, Madrid, Spain). After DNase I treatment, reverse transcription was performed with Superscript II reverse transcriptase (Invitrogen) to generate complementary DNA. The qRT–PCR was performed with an Applied Biosystems 7900 Real-time PCR System. In human lung specimens, the measurement of TMPRSS4 expression was carried out with the *TaqMan Gene Expression Assays* (Applied Biosystems, Foster City, CA, USA), according to the manufacturer's protocol. This plate contains the following human endogenous genes: *β-actin*, *HPRT* and *IPO8*. To normalise gene expression in the tissues samples, IPO8 was selected as an accurate control gene, as previously described (Nguewa *et al*, 2008). In the cell lines, RT–PCR reactions were carried out with SYBR Green PCR Master Mix (Applied Biosystems) and GAPDH levels were used as controls. The mean cycle threshold value (Ct) for the gene of interest normalised to the Ct value of the housekeeping gene was used to calculate gene expression values.

### Proliferation and clonogenic assays

To determine proliferation of H358, H441 and H2170 cells and their shRNA clones, 1200 cells for H358 and H441, and 2000 cells for H2170 per well were seeded in 96-well plates. After 96 hours of plating, MTT assays were performed (Roche, Palo Alto, CA, USA) following the manufacturer's protocol. To evaluate the clonogenic potential of H358 and H441 cells and their respective shRNA clones, 300 cells per well were plated into six-well plates. After 15 days of culture, colonies were fixed with 10% buffered formalin and stained with 2% crystal violet. The number of colonies was determined and data are presented as percentage with respect to parental cell line.

### Wound healing *in-vitro* migration assay

Cell migration was evaluated with an *in-vitro* model of wound healing. Cells were grown until confluence and monolayers were scraped with a 20-p micropipette tip. After 24 h, pictures of the wounds were taken with a Nikon Eclipse photomicroscope (Nikon, Kingston, UK) using the ACT-2U1.6 software (Nikon, Kingston, UK). The distance between the wound edges was measured with the Image J analysis software (NIH Image, Bethesda, USA). Six wells per condition were used.

### Animal model and histology

To determine the role of TMPRSS4 in lung metastasis, NOD SCID ILRγ2 (NSG) mice were used. All procedures were carried out in accordance with the guidelines for animal experimentation of our Institution (University of Navarra), under approved protocols.

In all, 5 × 10^5^ of H358 shCtrl, shTMP4-2 or shTMP4-3 cell clones were injected into the tail vein. To monitor lung metastasis by bioluminescence, mice were anaesthetised with a mixture of ketamine (150 mg kg^−1^) and xylazine (10 mg kg^−1^) intraperitoneally. Then, 1.5 mg D-luciferin in 100 *μ*l of PBS was injected. Imaging was completed at 2 min for each group of mice with a Xenogen IVIS system coupled to Living Image acquisition and analysis software (Xenogen Inc., Alameda, CA, USA). Photon flux was calculated for each mouse by using a circular region of interest for each lung. The background value (from luciferin-injected mice with no tumour cells) was subtracted from each measurement.

The lung tissues were fixed in 10% buffered formalin, embedded in paraffin and sectioned (5 *μ*m in thickness). Slides were stained with H&E, and histological images were captured with a Leica microscope (Wetzlar, Germany).

### Microarray data analysis and statistics

Data from the lung cancer microarrays previously described (Bild *et al*, 2006; Potti *et al*, 2006; Raponi *et al*, 2006; Lee *et al*, 2008; Shedden *et al*, 2008) were analysed. The statistical analyses were performed with GraphPad Prism 5.0 (GraphPad Software Inc., San Diego, CA, USA) and R 2.6.0 sofware (www.R-project.org/). Significant differences in TMPRSS4 gene expression between lung adenocarcinomas and squamous carcinomas or between the tumour and non-malignant specimens were analysed by unpaired two-tailed Student's *t*-test. Kaplan–Meier plots were used to illustrate differences in progression according to the level of TMPRSS4 gene expression. The TMPRSS4 expression was dichotomised using the tertile values (low expression included in tertile 1 and high expression in tertiles 2 and 3). Differences in overall survival (OS) (censored at 48 months) were compared using the log-rank test. *P*-values < 0.05 were considered statistically significant.

## Results

### TMPRSS4 is expressed in lung cancer cell lines

**Figure 1:**
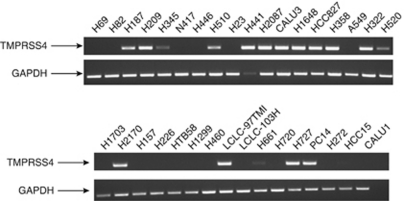
TMPRSS4 is expressed in lung cancer cell lines. The RT–PCR analysis for TMPRSS4 expression in 34 human lung cancer cell lines. In all, 17 cell lines were positive for this gene. The GAPDH was used as an internal control.

We first investigated the expression of TMPRSS4 in 34 human lung small cell lung cancer (SCLC) and NSCLC cell lines using RT–PCR. The TMPRSS4 mRNA was detected in half (17 out of 34) of the cell lines tested (Figure 1). The TMPRSS4 was highly expressed in 14 cell lines: Eight of them were derived from AC (H441, H2087, CALU3, H1648, HCC827, H358, PC14 and H322), one from SCC (H2170), three from SCLC (H187, H209 and H510), one from large cell carcinoma (97TM1) and one from carcinoid (H727). As TMPRSS4 had been linked to tumour development, we decided to study the role of this protein in lung cancer. To this end, we selected two cell lines derived from AC (H358 and H441) and one derived from SSC (H2170), with evident high expression of TMPRSS4 mRNA.

### TMPRSS4 inhibition reduces lung cancer cell proliferation and migration

**Figure 2:**
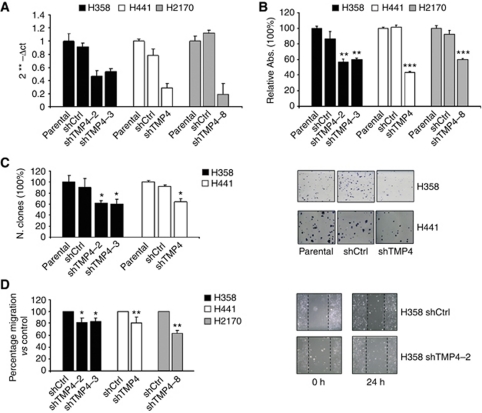
Knockdown of TMPRSS4 by shRNA reduces proliferation, clonogenic potential and migration of lung cancer cell lines. (**A**) Expression of TMPRSS4 in H358, H441 and H2170 cell lines after transfection with control shRNA or TMPRSS4-specific shRNA (shTMP4 for simplification), measured by qPCR. The GAPDH was used as internal control. (**B**) After 96 h of plating MTT assay was performed. An inhibition in the proliferation of H358, H441 and H2170 cells transfected with TMPRSS4-specific shRNA (*P*<0.01) was observed. (**C**) Clonogenic assay showed a lower number of clones in H358 and H441 shTMP4 cells compared with the control cells. (**D**) Migration of H358 (*P*<0.05), H441 (*P*<0.01) and H2170 (*P*<0.01) cells was impaired (about 20%) by the suppression of TMPRSS4. ^*^P<0.05; ^**^P<0.01; ^***^P<0.001.

To inhibit endogenous high TMPRSS4 levels, these three cell lines (H358, H441 and H2170) were transfected with control short-hairping RNA (shCtrl) or TMPRSS4-specific shRNA (shTMP4). As explained in Material and Methods, in the case of H358 and H2170 cells, some clones (shTMP4-2 and shTMP4-3 for H358 and shTMP4-8 for H2170) with a reduction higher than 60% in TMPRSS4 levels (Figure 2A) were selected and used for further experiments. For H441 cells, the whole cell population carrying the vector, with more than 70% reduction in TMPRSS4 expression was used (Figure 2A). This approach avoided a possible selection of particular clones with slow proliferation rates. Next, an MTT assay was carried out to assess the effect of TMPRSS4 knockdown on cell growth. As shown in Figure 2B, a shRNA-mediated depletion of TMPRSS4 resulted in a strong reduction of cell growth in clones from the three cell lines (*P*<0.01). In the clonogenic assay, we also observed a significant reduction in the number of colonies in H358 shTMP4-2 and shTMP4-3, and in H441-shTMP4 cells compared with the control cells (Figure 2C). Clonogenic experiments could not be performed with the H2170 cell line and its shRNA clones because, in our hands, these cells did not form colonies with a sufficient size so as to be accurately quantified. These data indicate that TMPRSS4 inhibition decreases cell growth in lung cancer cells.

We next investigated whether TMPRSS4 would modify unidirectional cell migration in the lung cancer cells. For this purpose, parental H358, H441 and H2170 cells or cells carrying the shRNA vectors were subjected to a wound-healing assay. As shown in Figure 2D, 24 h after plating, downregulation of TMPRSS4 caused a 20% reduction in the ability of H358, H441 and H2170 cells to migrate. Therefore, TMPRSS4 participates in the lung cancer cell migration.

### Downregulation of TMPRSS4 results in a decrease in lung metastasis

**Figure 3:**
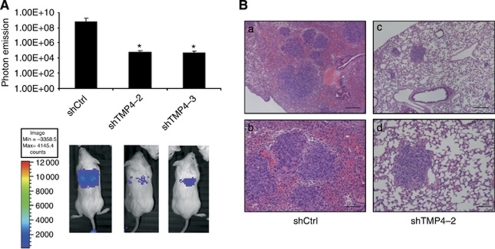
Downregulation of TMPRSS4 results in a decrease in lung tumour growth. (**A**) Representative luminometric images of mice injected with 5 × 10^5^ H358 control or shTMP4 cell clones in the tail vein. Photon emission quantification revealed a significant reduction in tumour growth in the groups injected with shTMP4-2 and shTMP4-3 cell clones (*P*<0.05). (**B**) Representative histological images of lungs from mice injected with H358 shCtrl (*a*, *b*) or shTMP4-2 (*c*, *d*) cell clones. Large tumours with highly angiogenic and haemorrhagic areas could be seen in control mice, but not in the shTMP4 group. Bars: *a, c*: 300 *μ*m; *b, d*: 120 *μ*m. ^*^P<0.05.

*In-vivo* experiments were performed to determine the role of TMPRSS4 in lung cancer development. The NSG mice were injected into the tail vein with 5 × 10^5^ H358 control cells, shTMP4-2 or shTMP4-3 cell clones. Bioluminescence image analysis was performed to measure tumour growth. The subsequent photon emission quantification indicated a significant reduction (*P*<0.05) of the tumour growth at day 10 after the cell injection (Figure 3A). At the end of the study (day 35), animals were killed and the lungs were examined for metastatic lesions. Metastatic foci in the lungs of all mice were evaluated by histological analysis (Figure 3B). In mice injected with H358 shCtrl cells, we observed an average of 20.17±9.94 metastatic foci per mice, while in mice injected with H358 shTMP4 cell clones we found 13.33±5.55 (shTMP4-2) and 13.86±14.6 (shTMP4-3). Although there were no statistical differences, low levels of TMPRSS4 tended to be associated with fewer metastatic foci per mice. Besides, large metastatic areas with highly angiogenic and haemorrhagic areas could be seen in control mice, but not in the shTMP4 group (Figure 3B). These results suggest a role of TMPRSS4 in lung tumour growth and metastasis formation.

### TMPRSS4 mRNA levels are dramatically increased in human lung cancer samples compared with non-malignant tissue, particularly in squamous cell carcinomas

**Figure 4:**
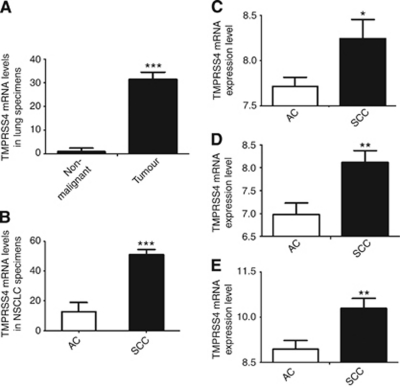
TMPRSS4 expression in clinical lung specimens. (**A**) TMPRSS4 levels were significantly higher (>30-fold increase, *P*<0.001) in tumours than in non-malignant samples, and (**B**) in SCC than in AC lung tumour specimens (*P*<0.001). (**C**–**E**) To validate these results, an analysis was carried out in three additional cohorts of patients. Again, tumours with SCC histology showed significantly more TMPRSS4 expression than tumours with AC histology, in these three different databases: (**C**) Potti *et al* (2006) (**D**) Lee *et al* (2008) and (**E**) Bild *et al* (2006). ^*^P<0.05; ^**^P<0.01; ^***^P<0.001.

The aforementioned results suggested a protumorigenic role of TMPRSS4 in lung cancer. Because a reliable antibody against TMPRSS4 is not yet available, we evaluated mRNA levels of this protease by qRT–PCR in a clinical sample set including normal (*n*=15) and NSCLC (*n*=30; 11 AC+18 SCC+1 sarcomatoid epidermoid carcinoma histology) samples. This cohort of patients included stage I–IV tumours (Table 1). Figure 4A shows that TMPRSS4 expression was dramatically upregulated (>30-fold) in lung tumours compared with normal lungs (*P*<0.001). In this cohort of patients, those with SCC histology showed significantly higher TMPRSS4 levels than those with AC histology (*P*<0.001) (Figure 4B).

In order to validate these results in larger and independent series of patients, we retrieved TMPRSS4 data from three different microarray expression databases with both AC and SCC cases (Bild *et al*, 2006; Potti *et al*, 2006; Lee *et al*, 2008). These databases included a total number of 420 NSCLC patients. No results from normal lung were available for any of the databases and only comparisons between TMPRSS4 expression in both the types of tumour histologies could be performed. Confirming our previous results, lung SCC exhibited significantly higher levels of TMPRSS4 than AC (Figure 4C–E) in the three databases.

### High mRNA levels of TMPRSS4 correlated with poor outcomes

**Figure 5:**
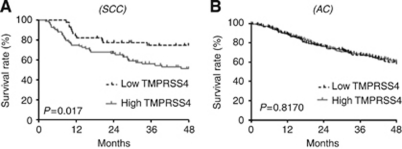
TMPRSS4 levels and clinical outcome. (**A**) High expression of TMPRSS4 significantly correlated with poor OS in lung SCC patients from Raponi *et al*, (*P*=0.017). (**B**) No association with OS in patients with AC histology was observed from the data published by Shedden *et al* (2008) (*P*=0.817).

We then investigated whether the expression of TMPRSS4 was associated with clinical outcome in NSCLC, depending on histology. For this purpose, we used databases with a large number of patients that specifically included either AC or SCC, where information on OS was available. Two databases, one for SCC (Raponi *et al*, 2006) and the other one for AC (Shedden *et al*, 2008) were used. In SCC, high levels of TMPRSS4 significantly correlated with reduced OS (Figure 5A, *P*=0.017). In the case of AC, consistent with the lower levels of TMPRSS4 found in patients (Figure 4), there was no discrimination between both of the subgroups (high and low mRNA TMPRSS4 levels) and prognosis (*P*=0.8170) (Figure 5B). We also analysed mRNA TMPRSS4 levels in relation to disease-free survival (DFS) in the AC series (Shedden *et al*, 2008), as data in SCC (Raponi *et al*, 2006) were not available. In agreement with the results found for OS in patients with AC histology, no statistical relationship was observed. We conclude that high TMPRSS4 mRNA levels are associated with poor prognosis in the NSCLC patients with SCC histology.

## Discussion

The present study reports the novel finding that TMPRSS4 is highly overexpressed in NSCLC and has a role in lung cancer development. We show here first evidence that TMPRSS4 is highly expressed in lung cancer cell lines and patients, and that such expression is significantly associated with poor prognosis in the case of SCC histology. Recent studies in thyroid, pancreatic and colon carcinoma showed that TMPRSS4 has a protumorigenic and metastatic role in these tumour types (Wallrapp *et al*, 2000; Kebebew *et al*, 2005). Because of the critical need for identifying new therapeutic targets for advanced lung cancer, and the fact that a previous study in colon cancer suggested that TMPRSS4 might also be involved in lung carcinogenesis, we hypothesised that this protease would increase the malignant behaviour of NSCLC cells. Analysis of the expression of TMPRSS4 mRNA levels in 34 lung cancer cell lines revealed that the majority of positive cells corresponded to AC histology. Three cell lines, two derived from AC (H358 and H441) and one derived from SSC (H2170) were selected for functional assays, as they showed high TMPRSS4 levels. Interestingly, depletion of TMPRSS4 in these cell lines using shRNA technology significantly affected their proliferation and clonogenic potential. These results indicate that this serine protease induces cell growth in NSCLC.

Metastatic implications of TMPRSS4 have been studied in colon cancer (Jung *et al*, 2008), which prompted us to analyse cell migration and metastasis *in vivo*. We have demonstrated that knockdown of TMPRSS4 by shRNA reduces significantly cell migration *in vitro* in H358, H441 and H2170 cell lines. Furthermore, in an *in vivo* model of lung cancer metastasis we show that inhibition of TMPRSS4 in luciferase-expressing H358 cells results in a decline in luminometric signals in the lungs. The TMPRSS4 downregulates epithelial markers, such as E-cadherin and P-cadherin, and upregulates mesenchymal markers that induce EMT in colon cancer cells (Jung *et al*, 2008). Our findings reinforce the main role of proteases (including TMPRSS4) in invasion and metastasis development.

We further evaluated TMPRSS4 expression in both the lung tumour and non-malignant samples. Tumour specimens exhibited much higher levels than normal lungs. In addition, we found that TMPRSS4 levels were higher in SCC patients as compared with those with AC histology, both in our series of patients as well as in microarray-retrieved data (Bild *et al*, 2006; Potti *et al*, 2006; Lee *et al*, 2008). This is a surprising result that contrasts with the predominant expression of TMPRSS4 in AC-derived cell lines. It is possible that the conditions of the tumour microenvironment switches on TMPRSS4 expression in SCC cells *in vivo* and that these conditions are not reproduced in *in vitro* cultures. Alternatively, stromal cells within the squamous tumour may be responsible for TMPRSS4 expression, instead of tumour cells themselves. Reliable antibodies should be developed in order to ascertain the precise location of TMPRSS4 in SCC tumours.

The next question we addressed was whether the expression of TMPRSS4 was associated with the clinical outcome in NSCLC, especially in SCC, where TMPRSS4 levels were found to be the highest. We hypothesised that an increase in this protease would worsen clinical outcome in the NSCLC patients. For this analysis, we used a database (Raponi *et al*, 2006) that includes a large and well-characterised series of SCC patients. Results indicate that high TMPRSS4 mRNA levels are significantly associated with poor prognosis for SCC. These results show for the first time (to the best of our knowledge) an association between high TMPRSS4 and poor prognosis.

All these data support our hypothesis that TMPRSS4 has an important role in lung cancer, promoting cell proliferation, tumour growth and invasion. Based on our results in patients, we propose TMPRSS4 as a putative biological marker for NSCLC (particularly for SCC histology), and as an indicator of poor prognosis. The development of specific antibodies (which are lacking at this moment) against this protease will allow further studies by immunohistochemistry in independent and larger series of patients to confirm our mRNA-based results. In addition, TMPRSS4 could also be considered as a therapeutic target. Although therapeutic efficacy of MMPs inhibitors rendered unsatisfactory results in clinical trials (Chu *et al*, 2007; Gialeli *et al*, 2011), it may not be the case for the family of serine proteases, including TMPRSS4. Future strategies for the development of TMPRSS4-targeting therapies (that may include blocking peptides or antibodies) should be explored. What seems clear is that the identification of squamous NSCLC-specific proteins (such as TMPRSS4) opens new avenues for diagnosis and treatment with this type of disease.

## Figures and Tables

**Table 1:** Clinical and pathological features of patients

**Patients**	**Sex**	**Age (years)**	**pTNM**	**Stage**	**Histology**
1	F	74	T2N1	IIB	Adenocarcinoma
2	F	54	T1N0	IA	Adenocarcinoma
3	M	70	T2N0	IB	Adenocarcinoma
4	M	77	T2N0	IB	Adenocarcinoma
5	M	63	T2N0	IB	Adenocarcinoma
6	M	71	T2N2	IV	Adenocarcinoma
7	M	56	T2N2	IIIA	Adenocarcinoma
8	M	60	T2N0	IB	Adenocarcinoma
9	M	77	T2N2	IIIA	Adenocarcinoma
10	M	54	T2N0	IB	Adenocarcinoma
11	F	62	T2N0	IB	Adenocarcinoma
12	M	78	T1N0	IA	Squamous carcinoma
13	M	49	T1N0	IA	Squamous carcinoma
14	M	66	T2N0	IB	Squamous carcinoma
15	M	59	T1N0	IA	Squamous carcinoma
16	M	79	T1N0	IA	Squamous carcinoma
17	M	63	T2N1	IIB	Squamous carcinoma
18	M	76	T2N1	IIB	Squamous carcinoma
19	M	77	T2N1	IIB	Squamous carcinoma
20	M	81	T2N0	IB	Squamous carcinoma
21	M	63	T2N0	IB	Squamous carcinoma
22	M	76	T2N0	IB	Squamous carcinoma
23	M	78	T2N1	IIB	Squamous carcinoma
24	M	75	T3N2	IIIA	Squamous carcinoma
25	M	76	T2N2	IIIA	Sarcomatoid epidermoid carcinoma
26	M	75	T3N1	IIIA	Squamous carcinoma
27	F	66	T2N0	IB	Squamous carcinoma
28	M	87	T2N0	IB	Squamous carcinoma
29	M	69	T2N2	IIIA	Squamous carcinoma
30	M	49	T1N2	IIIA	Squamous carcinoma
